# Case Report: Using Telehealth to Treat Triceps Tendinopathy in a Rock Climber

**DOI:** 10.3389/fspor.2022.829480

**Published:** 2022-03-21

**Authors:** Jared Vagy

**Affiliations:** Division of Biokinesiology and Physical Therapy, University of Southern California, Los Angeles, CA, United States

**Keywords:** physical therapy, elbow pain, regional interdependence, telehealth, rock climbing

## Abstract

This case study presents a 38-year-old, female rock climber with posterior elbow pain who was evaluated and treated using Telehealth. The use of telehealth for a clinical exam requires a larger emphasis be placed on posture observation and movement analysis since hands on assessment techniques cannot be used. During the patient exam, movement analyses identified scapulohumeral positional faults and dyskinesis, while self-palpation and self-midline resistance testing helped identify that the triceps tendon was the pathological tissue. A comprehensive rehabilitation program was developed based on concepts of regional interdependence to treat contributing factors in the scapular region and source tissues in the brachial region. After 10 weeks, the climber's pain decreased from 4/10 to 0/10. She made a full recovery back to her previous grade of V8 bouldering and was able to complete a V10 longstanding boulder project pain-free. This is the first case study of its kind to identify unilateral scapular dyskinesia in a patient with suspected triceps tendinopathy and to demonstrate a positive treatment effect by combining scapular strength exercises with eccentric exercises addressing the affected tissue.

## Introduction

Since COVID-19 emerged in the first months of 2020, social distancing and stay-at-home orders moved telehealth from a convenient option to an essential tool (Lee, 2020). As a result of nation and local mandates, many medical providers had to adjust their practice models to include telehealth care. Telehealth is performed in the field of physical therapy mostly through utilizing two-way synchronous audio and video. It has several strengths when compared to the in-person setting. Telehealth has the benefit of improved access to care (Branford et al., 2016; Seto et al., 2019), reduced travel time (Seto et al., 2019), improved convenience (Powell et al., 2017), improved patient engagement (Guo and Albright, 2017), reduced costs (Powell et al., 2017; Jiang et al., 2019; Seto et al., 2019), and improved session attendance (Kairy et al., 2009; Morris et al., 2011). Telehealth objective exam measures have been shown to be valid and reliable (Russell et al., 2010; Somerville et al., 2017) and interventions have been shown to improve pain and physical function (Cottrell et al., 2016). Telehealth has comparable patient satisfaction levels when compared to in-person rehabilitation (Moffet et al., 2017).

Although Telehealth presents some advantages when compared to in-person sessions it also has drawbacks. These include technology barriers (Lin et al., 2018; Seto et al., 2019), increased difficulty with exam measures (Powell et al., 2017), patient/provider preferences (Kruse et al., 2016), security, privacy, and confidentiality challenges (Hall and Mcgraw, 2014; Powell et al., 2017). However, most notably, since manual assessments cannot be performed by the clinician, the objective exam has a greater emphasis on movement analysis (Malliaras et al., 2021). Although this can be a potential barrier, it can also be viewed a benefit. By not being able to manual assess a patient, the clinician needs to rely more heavily on analyzing a climber's movement. This may allow them to look past the affected pain region and integrate concepts on regional interdependence (Wainner et al., 2007) into their diagnostic procedures. Regional interdependence is a concept that unrelated impairments in a remote anatomical region may contribute to or be associated with the patient's primary complaint. By using this concept remotely, clinicians may be able to uncover impairments that may have been missed in an in-person clinical exam that was solely focused on the painful region. Additionally, since it has been shown that telehealth improves patient self-efficacy (Guo and Albright, 2017), it can be utilized for improved patient engagement by placing a greater emphasis on the self-performance of corrective exercises and optimizing movement patterns.

This article focuses on a rock climber who was evaluated and treated using telehealth with suspected triceps tendinopathy. There are three heads of the triceps muscle: the medial, lateral, and long head. The three heads share a central tendon that inserts into the olecranon process of the elbow. Triceps tendinopathy, like other tendinopathies, occurs when repetitive use of the tendon leads to activation and proliferation, matrix changes including disorganization of collagen and neovascularization. In a prospective single-institution study that evaluated the demographics of 911 independent climbing injuries (Shöffl et al., 2015), the most 149 common body regions injured were the finger (52%), shoulder (17.2%), hand (13.1%), and the forearm and elbow (9.1%). Most of the elbow injuries in the study (5.5%) were identified as epicondylitis. Triceps tendinopathy is not only a rare condition in climbers but also in the general population with some studies citing a 3.8% prevalence of elbow injuries when assessed with MRI (Koplas et al., 2011). However, although prevalence is low, the methods used in this case study to assess the injury in the remote setting can be generalized to all body regions.

## Methods

**Figure 1:**
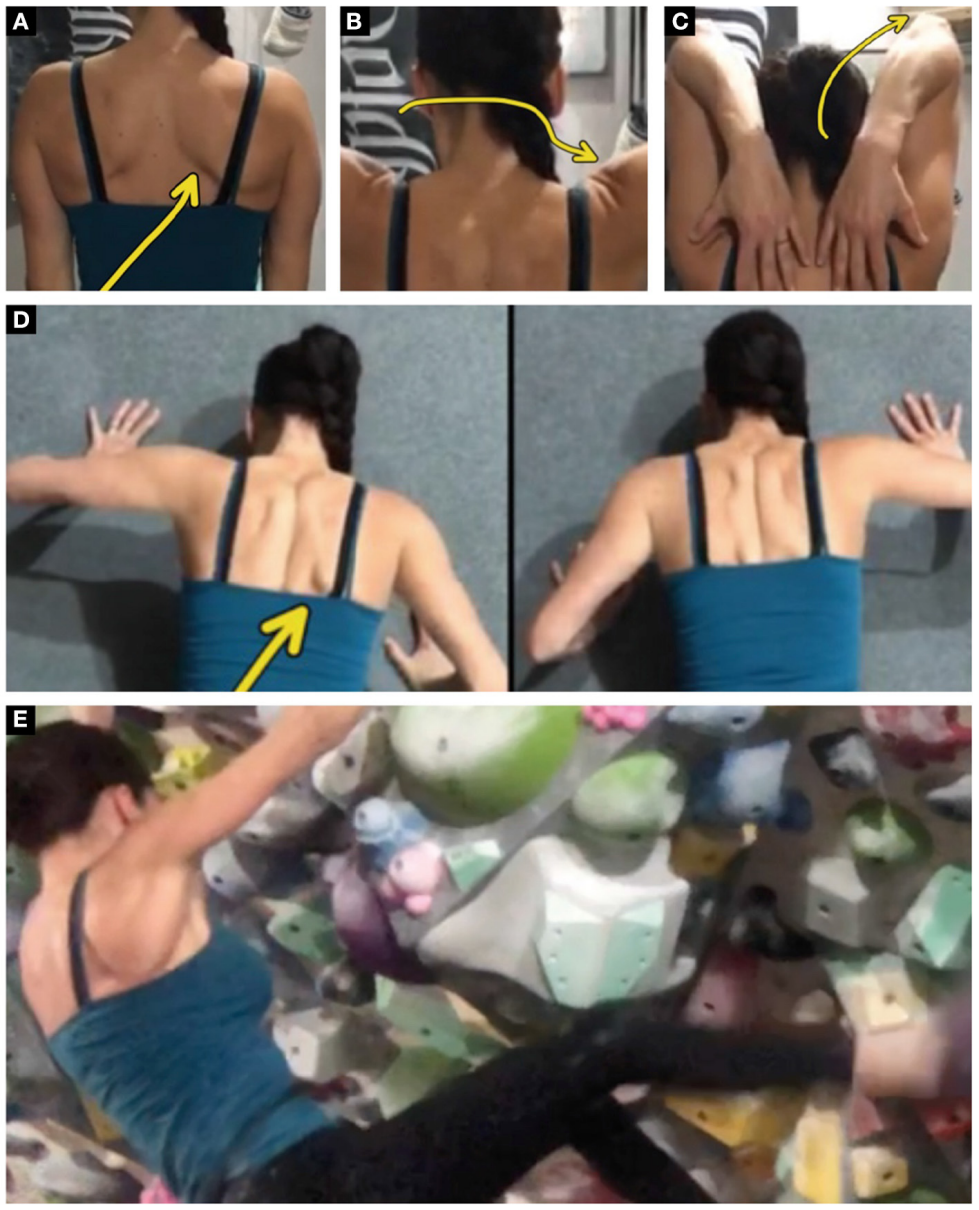
**(A)** Winging of the inferior angle of the scapula right greater than left indicating serratus anterior weakness. **(B)** Increased right sided humeral creasing with shoulder abduction indicating possible humeral hypermobility and inadequate scapular upward rotation and elevation. **(C)** Right humeral internal rotation with shoulder/elbow flexion indicating a latissimus dorsi mobility deficit. **(D)** Right sided scapular winging with offset pushup indicating serratus anterior muscle weakness. **(E)** Scapular winging with climbing movement on overhung wall indicating serratus anterior muscle weakness.

A 38-year-old, female rock climber was evaluated *via* Telehealth for elbow pain. She had 20 years of bouldering experience with a maximum grade of V11 and a pre-injury grade of V8. The evaluation was performed remotely utilizing Telehealth and the patient had no previous plan of care developed for her elbow pain. She presented 4-months post-partum with right sided posterior elbow pain superior to the olecranon. She reported a chronic history of mild elbow pain lasting over 1 year with a severity of 1 out of 10 on the VAS scale with 0 being “no pain” and 10 being “pain as bad as it could possibly be.” She continued to train for climbing during her pregnancy until the final 4 weeks and took 1 month off from training after her baby was born. In 3 months prior to the evaluation, after having her baby, she reported that she had increased the volume and intensity of her climbing training and her elbow pain had increased to 4/10 during specific movements such as push-ups, bench-press, and wiping counters. She reported hard bouldering moves such as lock-offs and gastons increased her symptoms. She also reported a minor right ring finger injury 6 weeks prior to the evaluation and an acute left ankle sprain from a bouldering fall 4 weeks prior to the evaluation. Secondary to pain she was limited climbing the grade of V6. She denied any numbness or tingling. The remote clinical examination included a posture analysis (Figure 1A) and a movement analysis of shoulder abduction (Figure 1B), shoulder flexion with elbow flexion (Figure 1C), offset pushups (Figure 1D), climbing movement (Figure 1E), and self-assessment of palpation and midline resistance testing. Posture and movement analysis demonstrated asymmetric scapulohumeral positional faults and dyskinesis including excessive scapular winging, inadequate scapular elevation/upward rotation, and excessive humeral internal rotation with shoulder flexion. Self-palpation reproduced symptoms with moderate pressure and midline resistance testing of elbow extension was provocative for symptoms at 90 degrees of shoulder flexion (4/10) and 0 degrees of shoulder flexion (2/10).

**Figure 2:**
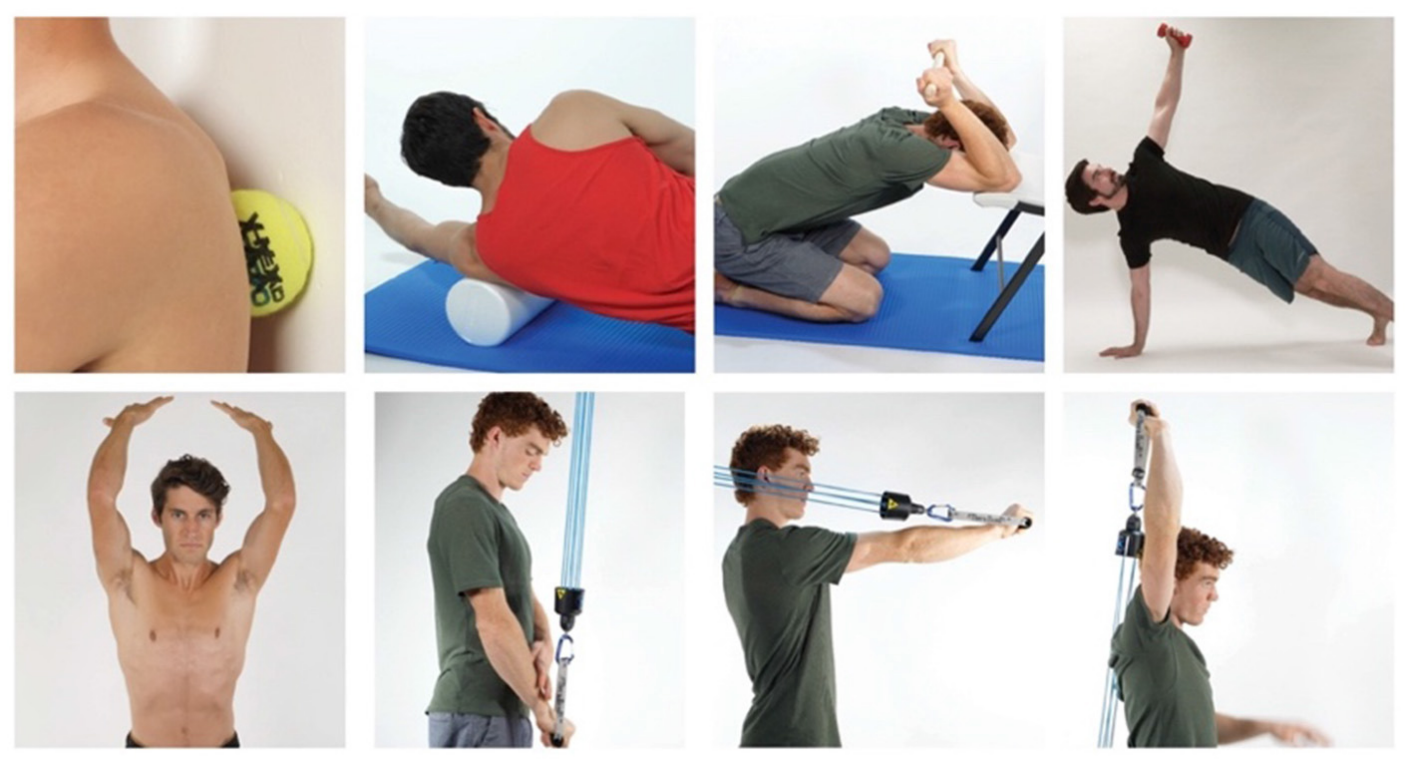
(From top left to bottom right) Tennis ball on posterior rotator cuff, latissimus dorsi soft tissue with foam roll, latissimus dorsi/triceps stretch with dowel, pushup-up plus airplane, wall taps, triceps eccentrics 0 degrees humeral flexion, triceps eccentrics 90 degrees humeral flexion, triceps eccentrics 180 degrees humeral flexion.

**Figure 3:**
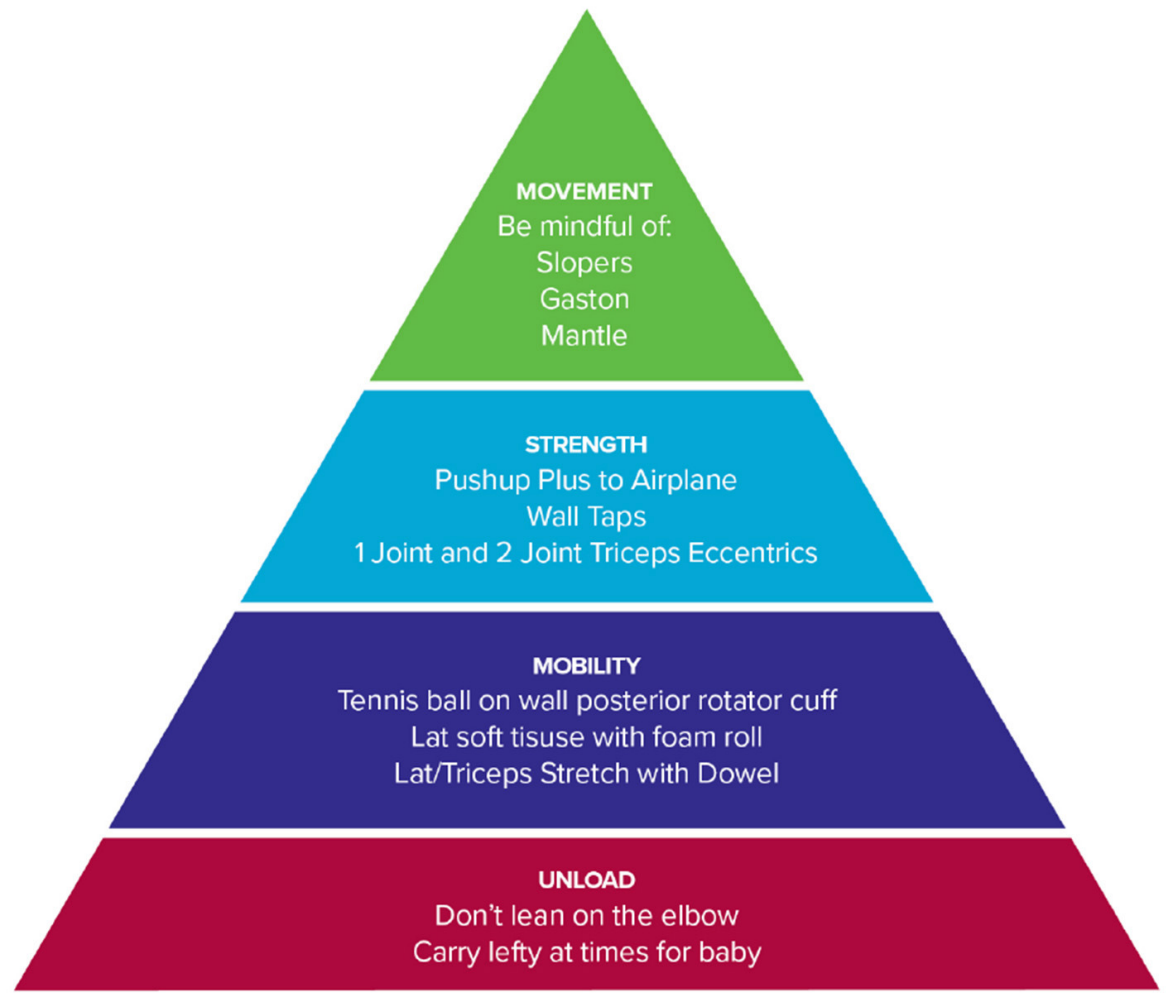
Organization of rehabilitation into a framework.

Based on the subjective reports and objective data gathered remotely, the patient was given a home exercise program based on a rehabilitation framework to unload the affected tissues, improve mobility, increase muscle performance, and retrain climbing movement. Each intervention was specially linked to a hypothesis driven movement impairment remotely tested during the session. Interventions consisted of patient education to carry her baby with her left arm and avoid leaning on the elbow. Her mobility exercises consisted of posterior rotator cuff and latissimus dorsi soft tissue mobilization followed by latissimus dorsi stretching. Each exercise was prescribed for 3 sets of 30 s daily. Her muscle performance exercises consisted of push-up plus airplane, wall taps, and triceps eccentrics performed at 0, 90 and 180 degrees of humeral flexion. Each exercise was prescribed for 3 sets of 8 repetitions to failure and performed 3 times per week. Adherence was assessed using a subjective report questionnaire. The patient was encouraged to return to her regular climbing schedule of 4 sessions per week (Figures 2, 3). The patient's goal for therapy was a single session remote evaluation with a self-administered home program. Exercises were reviewed during the initial evaluation with video and verbal feedback and correct exercise performance was confirmed. Detailed videos and written descriptions of the exercises were provided to the patient.

## Results

As a result of the remote environment, a large emphasis in the evaluation of the patient relied on subjective information and movement analysis. During movement analysis, it was discovered that the patient demonstrated scapulohumeral positional faults and dyskinesis including excessive scapular winging, inadequate scapular elevation/upward rotation, and excessive humeral internal rotation with shoulder flexion (Figure 1). The patient performed her home exercise program independently as prescribed for 10 weeks.

At 10-week follow-up the patient reported that her triceps pain was 0 out of 10 and she had returned to training and climbing pain-free at her previous grade of V8 without restriction. Additionally, she reported that 8 weeks after the evaluation she was able to complete a long-standing boulder project; a V10 problem called Sunshine which consists of challenging sloper and gaston moves (Table 1).

**Table 1:**
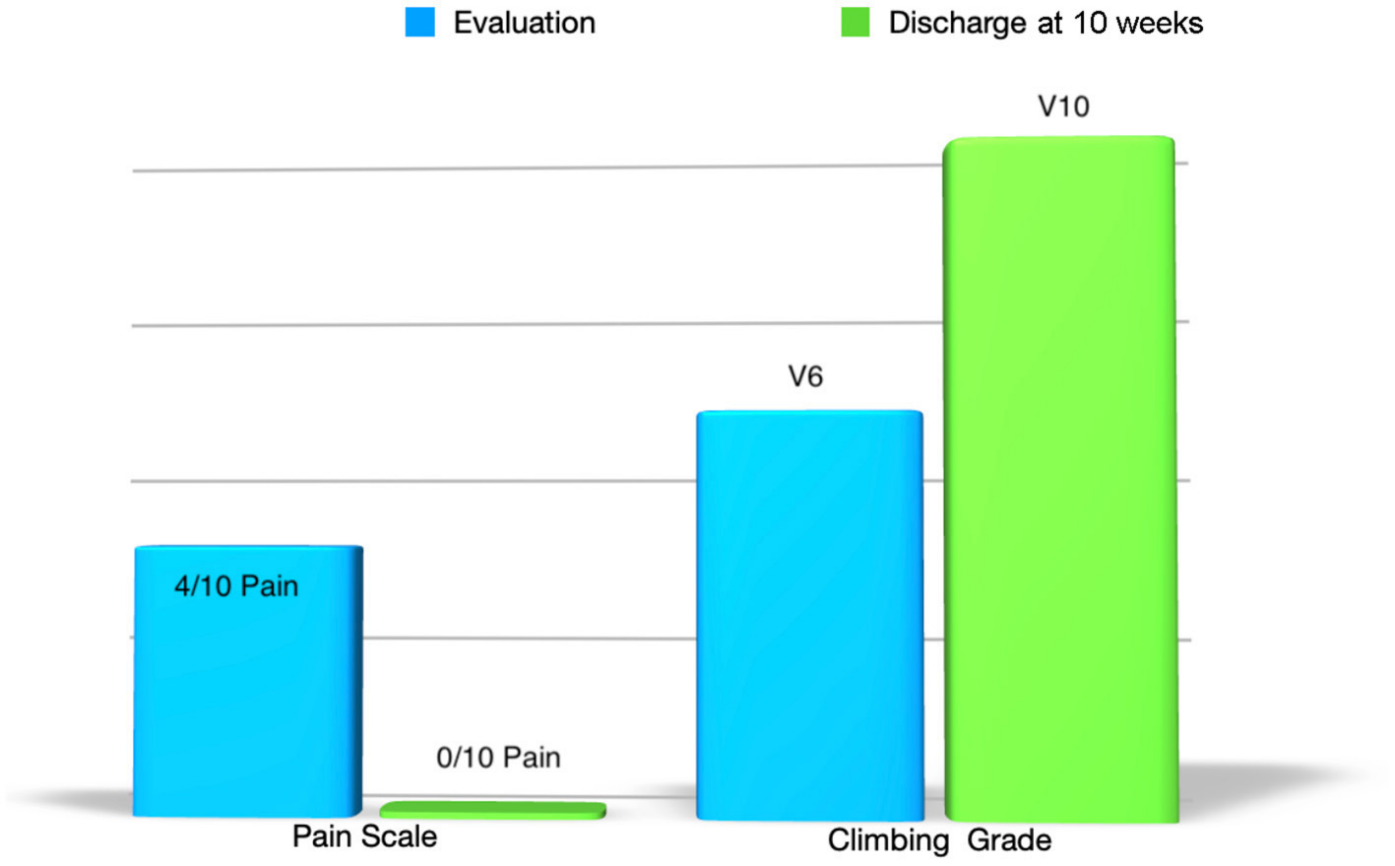
Pain levels and bouldering grade from evaluation to discharge.

## Discussion

There were benefits and drawbacks to evaluating this patient remotely. Since the patient worked full time and was caring for her child, the benefits of the session included reduced travel time and improved convenience. Since the session occurred during the early stages of the pandemic, the patient was able to minimize in-person contact risk by attending the session from the safety of her own home. Additionally, an added benefit was that the patient had a home climbing wall, and the clinician was able to observe climbing and exercises in the patient's home environment. The primary drawback of the Telehealth session was that many of the mobility and muscle performance deficits from the observed movement faults were built off hypothesis that could not be tested in the Telehealth setting. However, by not being able to perform manual tests and measures, it did allow for a greater emphasis to be paced on movement assessment. The emphasis on movement assessment uncovered some meaningful movement impairments that may have been missed during an in-person session.

The unloading techniques for the elbow included both carrying her baby with her opposite arm and not leaning on the elbow. The proposed theory for changing her carrying arm was to decrease the co-contraction of the triceps muscle needed to stabilize her baby while carrying. The theory behind not leaning on her elbow was to decrease potential irritation to the olecranon bursa (Reilly and Kamineni, 2016) which could further exacerbate the patient's triceps tendinopathy.

The rationale behind the mobility techniques targeting the periscapular musculature were directly related to the patient's observed impairments. Posterior rotator cuff mobility was prescribed with the hypothesis that a co-contracted infraspinatus and teres minor could lead to a lack of glenohumeral dissociation and contribute to scapular winging (Figure 1E). Latissimus Dorsi mobility was prescribed based on the movement observation excessive humeral internal rotation with shoulder flexion and elbow flexion (Figure 1C). The humeral internal rotation movement with end range shoulder flexion can lead to increased triceps contraction while climbing secondary to the altered elbow position.

It has been shown in research that scapular positioning and dyskinesis affect the elbow and when addressed can resolve elbow pain (Bhatt et al., 2013). Based on the concept of regional interdependence, scapular positioning and strengthening exercises were selected to treat the patient's elbow pain. And since it has also been shown that the push-up plus exercise elicits high levels of electro-myographic activity from the serratus anterior (Decker et al., 1999), a push-up plus variation was included in the treatment program. It has also been shown that eccentric exercises can have positive effects on pain and muscle strength in patients with lateral elbow tendinopathy (Yoon et al., 2021), and even more recently it has been shown that eccentric-concentric training combined with isometric contractions is an effective treatment for lateral elbow tendinopathy (Stasinopoulos and Stasinopoulos, 2017). The patient was prescribed eccentrics based on patient preference and supporting research.

The movement exercises were identified to decrease the overuse of the triceps and minimize scapular dyskinesia while climbing. The excessive use of gastons and mantles places a high degree of stress on the triceps musculature and the use of slopers exaggerated the climber's scapular winging, so a recommendation was made to avoid routes with the excessive use of slopers, gastons, and mantles.

Limitations of this case report include the lack of a standardized subjective report questionnaire at initial evaluation and follow-up, and the inability to achieve in-person objective tests and measures secondary to the remote environment.

## Conclusion

The use of telehealth to conduct a clinical exam requires that a larger emphasis be placed on posture observation and movement analysis since hands on assessment techniques cannot be used. Since rock climbing is a sport that requires precise movement performance, Telehealth can serve as a valuable tool to assess climbers with injuries especially when barriers exist to performing examinations in-person. Even in rare and difficult to diagnose conditions such as triceps tendinopathy, Telehealth can be an effective tool for clinical assessment and can be used to a return a climber back to optimal function.

## Data Availability Statement

The original contributions presented in the study are included in the article/supplementary material, further inquiries can be directed to the corresponding author/s.

## Ethics Statement

Ethical review and approval was not required for the study on human participants in accordance with the local legislation and institutional requirements. The patients/participants provided their written informed consent to participate in this study. Written informed consent was obtained from the individual(s) for the publication of any potentially identifiable images or data included in this article.

## Author Contributions

The author confirms being the sole contributor of this work and has approved it for publication.

## Conflict of Interest

The author declares that the research was conducted in the absence of any commercial or financial relationships that could be construed as a potential conflict of interest.

## Publisher's Note

All claims expressed in this article are solely those of the authors and do not necessarily represent those of their affiliated organizations, or those of the publisher, the editors and the reviewers. Any product that may be evaluated in this article, or claim that may be made by its manufacturer, is not guaranteed or endorsed by the publisher.
